# Identification and Characterization of Besifovir-Resistant Hepatitis B Virus Isolated from a Chronic Hepatitis B Patient

**DOI:** 10.3390/biomedicines10020282

**Published:** 2022-01-26

**Authors:** Jong Chul Kim, Hye Young Lee, Ah Ram Lee, Mehrangiz Dezhbord, Da Rae Lee, Seong Ho Kim, Juhee Won, Soree Park, Na Yeon Kim, Jae Jin Shin, Sang Gyune Kim, Young Seok Kim, Jeong-Ju Yoo, Kyun-Hwan Kim

**Affiliations:** 1 Department of Precision Medicine, School of Medicine, Sungkyunkwan University, Suwon 16419, Korea; rlawhdcjf95@naver.com (J.C.K.); ahram2g@naver.com (A.R.L.); asal@skku.edu (M.D.); edal_0422@naver.com (D.R.L.); seongho_0809@naver.com (S.H.K.); 1wonjuhee@hanmail.net (J.W.); rhd37@naver.com (S.P.); michaela3310@naver.com (N.Y.K.); jaejin362@kakao.com (J.J.S.); 2 Divsion of Gastroenterology and Hepatology, Department of Internal Medicine, School of Medicine, Soonchunhyang University, Jomaruro 170, Bucheon 14584, Korea; essen49@naver.com (H.Y.L.); mcnulty@schmc.ac.kr (S.G.K.); liverkys@schmc.ac.kr (Y.S.K.)

**Keywords:** hepatitis B virus, besifovir dipivoxil maleate (BSV), nucleos(t)ide analog, drug resistance, reverse transcription

## Abstract

Hepatitis B virus (HBV) is known to cause severe liver diseases such as acute or chronic hepatitis, liver cirrhosis and hepatocellular carcinoma. Chronic hepatitis B (CHB) infection is a major health problem with nearly 300 million individuals infected worldwide. Currently, nucleos(t)ide analogs (NAs) and interferon alpha are clinically approved treatments for HBV infection. NAs are potent antiviral agents that bind to HBV polymerase and block viral reverse transcription and replication. Besifovir dipivoxil maleate (BSV) is a newly developed NA against HBV in the form of acyclic nucleotide phosphonate that is available for oral administration similar to adefovir and tenofovir. Until now, resistance to BSV treatment has not been reported. In this study, we found a CHB patient who showed viral breakthrough after long-term treatment with BSV. The isolated HBV DNA from patient’s serum were cloned into the replication-competent HBV 1.2 mer and the sequence of reverse transcriptase (RT) domain of HBV polymerase were analyzed. We also examined the drug susceptibility of generated clones in vitro. Several mutations were identified in HBV RT domain. A particular mutant harboring ten RT mutations showed resistance to BSV treatment in vitro. The ten mutations include rtV23I (I), rtH55R (R), rtY124H (H), rtD134E (E), rtN139K (K), rtL180M (M), rtM204V (V), rtQ267L (L), rtL269I (I) and rtL336M (M). To further identify the responsible mutations for BSV resistance, we performed in vitro drug susceptibility assay on several artificial clones. As a result, our study revealed that rtL180M (M) and rtM204V (V) mutations, already known as lamivudine-resistant mutations, confer resistance to BSV in the CHB patient.

## 1. Introduction

HBV is a major cause of chronic liver diseases with estimated 1.5 million new infections each year (WHO 2019). Liver cirrhosis and hepatocellular carcinoma (HCC) are associated with more than 800,000 annual deaths worldwide [1].

HBV polymerase has different functions such as pgRNA packaging, protein priming, and DNA synthesis. HBV replication process initiates after pgRNA encapsidation and recognition of epsilon in the 5’ end of pgRNA by Pol (polymerase or reverse transcriptase). The pgRNA is then reverse transcribed by polymerase that is also packaged along with pgRNA inside the nucleocapsids. The HBV Pol contains a reverse transcriptase (RT) domain in which drug-resistance-related mutations or “YMDD motif variants” emerge in some CHB patients after prolonged Nucleos(t)ide analogs (NAs) treatment [2,3]. Inhibiting the reverse transcription process by NAs eventually suppresses HBV replication and prevents virion generation. More specifically, NAs inhibit HBV replication by competitively binding to the viral polymerase and terminating DNA chains. NAs obstruct 3’ hydroxyl groups of deoxyribonucleic acid during the elongation of HBV DNA, leading to the failure in synthesis of the nascent DNA molecule [4,5,6,7,8,9]. Various structures of NAs have been designed which specifically target certain viral polymerases. They compete with dATP, dTTP, dCTP, and dGTP substrates required for DNA or RNA synthesis, thereby inhibiting the replication of several other viruses such as HSV and HIV.

NAs and interferon (IFN)-α treatment are currently used to treat CHB patients. NAs including lamivudine (LAM), telbivudine (LdT), adefovir (ADV), tenofovir (TDF or TAF), and entecavir (ETV) [9,10,11]. Besifovir dipivoxil maleate (BSV) which is in the form of an acyclic nucleotide phosphonate similar to adefovir and tenofovir, has a broad spectrum of antiviral activity. The active form of BSV, guanosine monophosphate, undergoes phosphorylation in the hepatocytes where it competes with dGTP in binding to RT, terminates the DNA chain and thereby inhibits HBV replication.

The decision to initiate treatment for HBV patients is primarily based upon the presence or absence of cirrhosis, the ALT level as well as the HBV DNA level. For those who require treatment, tenofovir disoproxil fumarate (TDF), tenofovir alafenamide fumarate (TAF) or ETV are acceptable options in patients with treatment-naïve CHB [9,10,11,12].

Since May 2017, BSV was approved as a new NA drug in South Korea to treat naïve CHB patients. [13,14,15,16]. BSV demonstrated the same antiviral property as ETV (phase II clinical trial) or TDF (phase III clinical trial) with fewer side effects such as renal dysfunction or osteoporosis [14,17,18]. Considering these promising results, BSV is also recommended by the clinical practice guidelines of the Korean Association for the Study of the Liver [12].

As BSV is a relatively new anti-HBV agent with shorter clinical usage, no BSV-resistant data have been released so far [18]. Due to the both viral persistence and heterogeneity of the HBV genome, the emergence of drug-resistant mutants is inevitable. Recently, we found a BSV-resistant CHB patient after 64-weeks of BSV administration. Therefore, the purpose of this study was to identify the BSV-resistant HBV in a CHB patient and characterize the responsible mutations conferring resistance to BSV.

## 2. Materials and Methods

### 2.1. Patient

The serum samples of the CHB patient were received from Bucheon Soonchunhyang University Hospital. The study protocol was confirmed by the ethical guidelines of the World Medical Association Declaration of Helsinki and was approved by the Institutional Review Boards of Soonchunhyang University Bucheon Hospital (IRB number: SCHBC 2021-11-007, approval date: 9 November 2021). The patient was provided with the informed consent for this study.

### 2.2. HBV RT Sequence Analysis

Serum from CHB patient was collected at the indicated time to analyze HBV variants during the BSV administration period. Viral DNA was extracted using QIAmp MinElute Virus Spin Kit (QIAGEN, Hilden, Germany) according to the manufacture’s protocols. Using viral DNA as template, PCR was performed to specifically amplify the RT region. Primer sequences are indicated in Table S1, and conditions for amplification were as follows; 94 °C for 5 min followed by 30 cycles of 94 °C for 30 s, 65 °C for 30 s, 72 °C for 1 min, and 72 °C 10 min.

Next, amplified PCR products were recovered by gel extraction, and cloned into the pGEM-T vector (pGEM-T vector Systems, Promega, Madison, WI, USA). More than 10 clones were selected and sequenced (Figure S1). Mutations in the RT sequence of the selected clones were compared to a reference HBV sequence (wild-type (WT) genotype C, National Center for Biotechnology Information (NCBI) accession number: GQ872210).

### 2.3. Construction of HBV Reverse Transcriptase (RT) Mutant Replicons

The patient-derived or artificially generated HBV RT genes were amplified by PCR and cloned into HBV 1.2 mer replicon by infusion-cloning. As previously described [19], HBV 1.2 mer replicon were constructed using linearized vector and insert fragments by the NEBuilder HiFi DNA assembly Cloning Kit (New England Biolabs, Ipswich, UK) according to the manufacturer’s instructions.

### 2.4. Cell Culture, Transfection, and Drug Treatment

Huh7, human hepatoma cell line, was cultured in Dulbecco’s Modified Eagle’s Medium (Welgene, Gyeongsan-si, Korea) supplemented with 10% fetal bovine serum (Capricorn, Ebsdorfergrund, Germany) and 1% penicillin/streptomycin (Gibco, Carlsbad, CA, USA) at 37 °C in a humidified 5% CO_2_ incubator. The cells were seeded into 6 well plates and 2 μg of HBV replicons were transfected by Lipofectamine 2000 reagent (Invitrogen, Carlsbad, CA, USA). 5 h after transfection, the cell media was replaced with the media containing the indicated concentrations of besifovir (BFV). Drug was daily administrated in fresh medium. Four days after transfection, cells and supernatant were harvested to measure the levels of viral DNA and secreted antigens by Southern blot and ELISA, respectively.

### 2.5. Southern Blot Analysis

As described in our previous studies [19], southern blot was performed to analyze HBV replication. After 4 days of transfection, the cells were harvested and lysed with 100 μL of HEPES lysis buffer (10 mM HEPES, 100 mM NaCl, 1 mM EDTA, 1% NP-40) on ice, and after centrifugation, the supernatant was treated with Nuclease buffer I (10 mM CaCl_2_, 12 mM MgCl_2_, and 10 units of DNase I (Roche, Mannheim, Germany)) to digest the transfected plasmid DNA at 37 °C for 3 h. Then, 7.4% PEG (polyethylene glycol 8000, Sigma, St. Louis, MO, USA) was added to precipitate HBV capsids, followed by incubation overnight at 4 °C. To completely remove the remaining plasmid DNA, the PEG precipitates were resuspended with Nuclease buffer II (10 mM Tris-HCl, 8 mM CaCl_2_, 6 mM MgCl_2_, 10 units of DNase I) and incubated at 37 °C for 1 h. Next, in the presence of 0.5% sodium dodecyl sulfate (SDS), 240 ug/mL proteinase K (Roche) was added to break the HBV capsids and release HBV DNA. This was followed by phenol/chloroform/isoamyl alcohol (25:24:1), PCI purification and precipitation with 100% ethanol/3 M sodium acetate. The purified total HBV DNA was electrophoresed in 1% agarose gel (LE, analytical grade, Promega) and transferred onto the Hybond-N+ nylon membrane. Based on the HBV DNA full sequence, a DIG-labeled probe was designed and synthesized to target a total of 7 regions of HBV genome, using the PCR DIG probe Synthesis Kit (Roche), and HBV DNA was visualized using the generated probe [20]. The signals were analyzed by ImageQuant 800 (Amersham)

### 2.6. Enzyme-Linked Immunosorbent Assay (ELISA)

Before harvesting the cells, culture supernatants were diluted to 20-fold for HBeAg and 80-fold and HBsAg, respectively. The levels of secreted HBeAg and HBsAg were analyzed using ELISA kit (Wantai, Beijing, China) according to the manufacturer’s instructions. The optical density (OD) values were measured at 450 nm wavelength with a spectrophotometer (SpectraMax Plus 384).

### 2.7. Quantitative Real-Time PCR (RT-qPCR)

Quantitative real-time PCR was used to quantify the relative amount of intracellular HBV DNA. 5 μL of DNA prepared from southern blot was diluted 10-fold and 2 μL was used for PCR. The PCR mixture contained total of 15 μL including SYBR green master mix (Applied Biosystem) 7.5 μL, and 0.4 μM of each primer. Amplification conditions are as follows: 95 °C for 10 min, followed by 40 cycles of 95 °C for 15 s, and 60 °C for 1 min in QuantStudio 3 Real-Time PCR System (Applied Biosystem). Relative replication levels were calculated by the comparative 2^−∆∆CT^ method [19,21,22].

### 2.8. Statistical Analysis

At least three independent experiments were performed for each data analysis. Data are exhibited with mean± SD. Statistical analysis were performed using one-way ANOVA, * *p* < 0.05; ** *p* < 0.01; *** *p* < 0.001.

## 3. Results

### 3.1. Mutation Profile of HBV RT Domain Isolated from a Patient Treated with BSV

**Figure 1:**
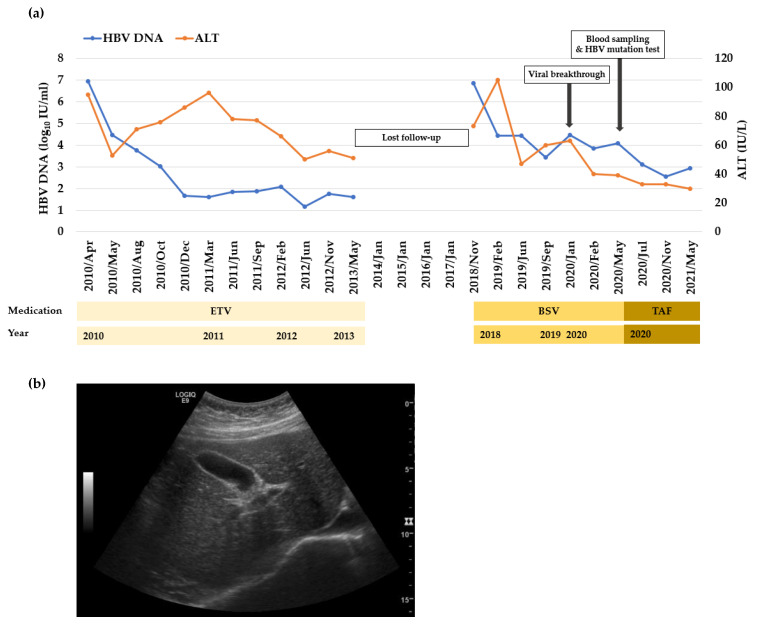
Clinical course and liver ultrasonography of the CHB patient with incomplete virological response during BSV treatment. (**a**) Clinical course during treatment of BSV. The patient first was treated with BSV in December 2018. In May 2020, drug regimen was changed from BSV to TAF. Serum samples were analyzed for HBV-DNA and ALT measurement. The time points of serum sampling are indicated by arrows. ETV, entecavir; BSV, besifovir dipivoxil maleate; TAF, tenofovir alafenamide fumarate. (**b**) The abdominal ultrasound showed evidence of early liver cirrhosis.

Clinical course of the subject CHB patient (female, 55-year-old) indicating incomplete virological response during BSV administration from December 2018 to March 2020 is profiled in Figure 1a. The patient was treated with ETV for 3 years between April 2010 and May 2013. Afterwards, the patient stopped antiviral treatment voluntarily, and was lost to follow-up after October 2013.

In December 2018, the patient began to receive BSV as the second line of anti-HBV therapy. Considering the safety aspect of drug treatment, it was preferable to alternate the antiviral regimen to a nucleotide analogue (BSV) as the patient was administered with a nucleoside analogue (ETV) in her medication history. The blood work results are listed in Table 1 and Figure 1a. Abdominal ultrasonography showed early liver cirrhosis (Figure 1b), and the result of transient elastography (Fibroscan^®^) was 12.0 kPa. Based on current guideline, the patient started BSV monotherapy (150 mg/day) from December 2018. After 17 months, viral breakthrough developed, and HBV DNA reached to 12,166 IU/mL with ALT level at 63 IU/L. HBV blood sampling was done at indicated time point for in vitro mutation analysis (Figure 1a). In May 2020, the BSV was switched to TAF, and HBV DNA was isolated from patient serum to identify the mutations in the RT domain of HBV polymerase which may confer resistance to BSV. From the total 16 obtained clones, 5 clones (Clone 1-1, 1-14, 1-19, 1-21, and 1-22) showed the same mutation profile, indicating that this clone is major in patient’s serum (Table 2). When compared to the WT, Clone 1-1 harbored 10 mutations (IRHEKMVLIM: rtV23I (I), rtH55R (R), rtY124H (H), rtD134E (E), rtN139K (K), rtL180M (M), rtM204V (V), rtQ267L (L), rtL269I (I) and rtL336M (M)). Clone 1-3 and 1-4 also exhibited similar mutation profile. Therefore, 43.8 % (7/16 clones) of obtained clones commonly harbored IRHEKMVLIM mutations.

Based on these results, we hypothesized that the IRHEKMVLIM mutations are probably associated with BSV resistance.

### 3.2. Patient-Derived HBV RT Mutant Harboring IRHEKMVLIM Is Resistant to BFV Treatment

BSV is an orally available drug which is hydrolyzed by esterase in the liver and intestine where the acetyl group is separated and converted into besifovir (BFV, active metabolite, LB80317). It is further phosphorylated in liver cells and competes with dGTP in binding to the HBV RT. Due to lack of converting enzymes in vitro, BFV was used to perform the drug susceptibility assays.

**Figure 2:**
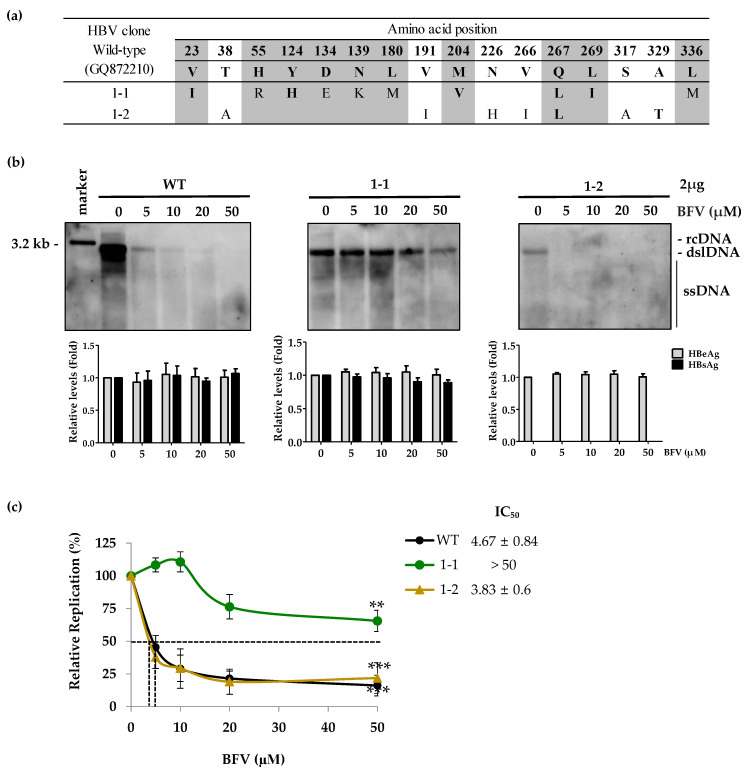
BFV susceptibility of the HBV RT mutants isolated from the CHB patient. (**a**) The amino acid sequences of HBV RT domains isolated from the CHB patient were analyzed by cloning and sequencing. (**b**) The HBV 1.2 mers cloned using the patient-derived RT domain were transfected into the Huh7 cells. HBV DNA and antigen levels were measured by Southern blot and ELISA, respectively. The sensitivity of Huh7 to BFV was determined by southern blot using an HBV-specific digoxigenin (DIG) labeled probe. The 3.2 kb marker is shown in lane 1 (**c**) IC_50_ values were determined by quantitative real-time PCR. ** *p* < 0.01; *** *p* < 0.001. Data were obtained from at least three independent experiments (mean ± SD). RT, reverse transcriptase; WT, wild type; BFV, besifovir.

To investigate which mutations are involved in BFV resistance, we constructed HBV 1.2mer replicons where the RT domain of WT was changed to that of patient-derived HBV Pol mutants. We selected Clone 1-1 and Clone 1-2 as the first and second most frequently identified clones respectively, for further experiments. Clone 1-2 harbored 7 mutations (AIHILAT: rtT38A (A), rtV191I (I), rtN226H (H), rtV266I (I), rtQ267L (L), rtS317A (A) and rtA329T (T)) (Figure 2a).

To evaluate the in vitro BFV susceptibility of RT mutants obtained from the subject, we transfected each patient-derived clone into the Huh7 cells. The levels of HBV DNA and antigens (HBeAg, HBsAg) were analyzed by Southern blot and ELISA, respectively (Figure 2b). Clone 1-1 showed strong resistance to BFV treatment, while WT was susceptible to dose-dependent treatment of BFV. Although the replication capacity of Clone 1-2 was lower than other clones, it showed similar BFV susceptibility as WT. Since NAs only inhibit reverse-transcription of pgRNA, the HBV antigen levels was not affected following BFV administration. Moreover, the constant HBeAg and HBsAg levels in each sample indicated the reliable transfection efficiency among all experimental sets. As shown in Table 3, the HBsAg level of Clone 1-2 was not detected due to the overlapping mutations which introduced two stop codons in the corresponding surface antigen gene.

As the replicative capacity of Clone 1-2 was too low for quantitative analysis by Southern blot, IC_50_ values were further determined through quantitative real-time PCR, which is a more sensitive method for analyzing HBV DNA levels. IC_50_ values for WT, Clone 1-1, and Clone 1-2 were 4.67 ± 0.84, >50, and 3.83 ± 0.6 μM, respectively (Figure 2c). The fold difference between IC_50_ values of WT and Clone 1-1 was over 10.7-fold. These results demonstrated that Clone 1-1 was the dominant HBV mutant in CHB patient harboring IRHEKMVLIM mutations and was strongly resistant to BSV.

### 3.3. HBV RT rtL180M and rtM204V Mutations Are Associated with BFV Resistance

**Figure 3:**
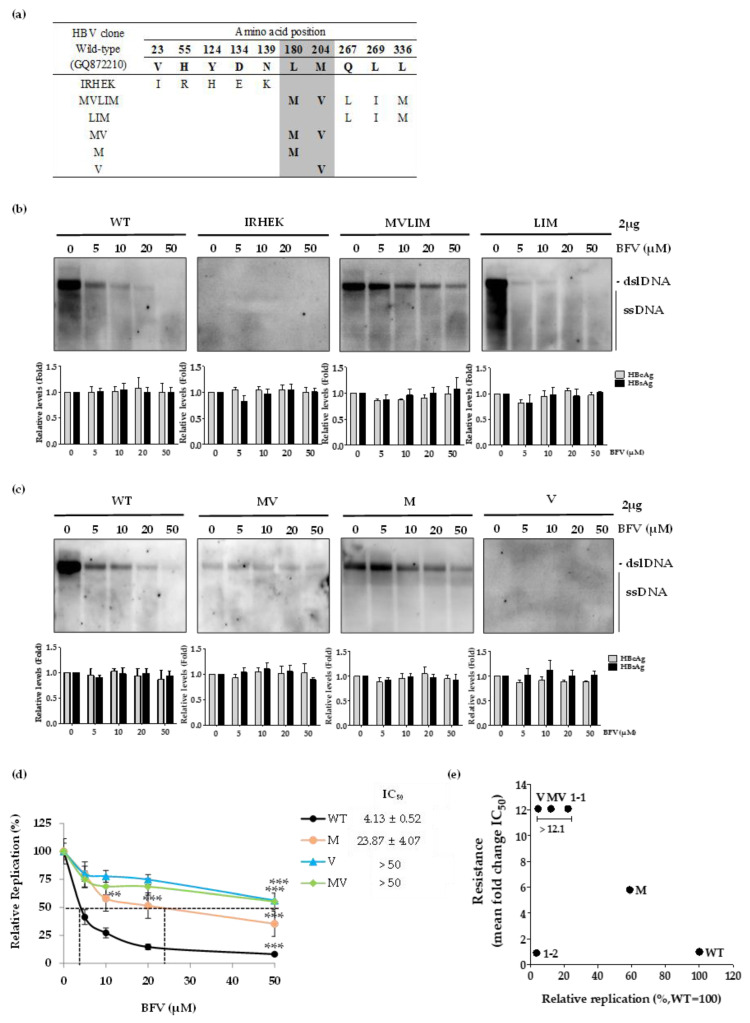
Identification of BFV-resistant mutation in patient-derived HBV mutant. (**a**) The sequences of patient-derived and artificial replicons constructed in this study are compared with that of WT. (**b**,**c**) The constructed HBV 1.2mer replicons were transfected into Huh7 cells, and BFV was treated every day in a dose-dependent manner as indicated. At 5 h post-transfection, BFV was treated for 3 days. The level of HBV DNA and secreted antigen were determined Southern blot and ELISA, respectively. ELISA was performed to confirm the transfection yield. (**d**) IC_50_ values were measured by quantitative real-time PCR. ** *p* < 0.01; *** *p* < 0.001. The level of HBV replication without drug treatment was set to 100 %. (**e**) Replication ability and BFV resistance of each clone were compared with WT through real-time PCR. All data were obtained from at least 3 independent experiments (mean ± SD).

To identify which mutations are responsible for BFV resistance in Clone 1-1, we constructed a series of replication-competent HBV 1.2 mer mutant clones as shown in Figure 3a. First, in order to determine the BFV susceptibility, 10 identified mutations (IRHEKMVLIM) were divided into two parts, i.e., IRHEK and MVLIM, and were cloned into two separate HBV replicon constructs (Figure 3a). The primers used to amplify specific RT region were listed in Table S1. Since the previous reports showed that two mutations (rtL180M or rtM204V) are associated with resistance to NA drugs such as LMV and ETV [23,24,25], the MVLIM mutant clone were divided into four distinct clones (LIM, MV, M, and V) to further screen the mutation that is responsible for BFV resistance.

Drug susceptibility assays demonstrated that the clone containing MVLIM mutations was considerably resistant to BFV treatment as compared to WT, whereas LIM clone was highly susceptible to BFV. Interestingly, a clone harboring IRHEK showed very low replication capability (Figure 3b, upper panel). Transfection yield of HBV replicons was constant because there was no significant difference in the secreted HBeAg and HBsAg levels in each well (Figure 3b, bottom panel).

Clones harboring MV or M mutation exhibited resistance to BFV treatment (Figure 3c). MV clone was more resistant to BFV compared to the M clone, while its replication capability was lower than that of M clone. The replication of V clone was barely detected by Southern blot due to its low replication capability. When the rtM204V mutation was accompanied by rtL180M, the replicative capacity was increased, demonstrating the complementary function of rtL180M mutation. This phenomenon is consistent with previous report, demonstrating that the rtL180M mutation in HBV polymerase is commonly accompanied by rtM204V or rtM204I mutations [23,26,27]. The relative replication levels of each clone were determined by quantitative real-time PCR (Figure 3d) and calculated IC_50_ values were summarized in Table 4. IC_50_ values for WT, M, V and MV were 4.13 ± 0.52, 23.87 ± 4.07, >50, and >50 μM, respectively. Relative to the IC_50_ value of WT, the fold differences in IC_50_ of M, V and MV clones were 5.8-fold, >12.1-fold, and >12.1-fold, respectively. The effect of each mutation on BFV resistance and replication ability obtained from real-time PCR, was compared with WT. The fold resistance of V, MV, and 1-1 clones was 12.1 times higher than that of WT (Figure 3e).

Taken together, these observations indicated that rtL180M and/or rtM204V substitution contributed to development of BFV resistance. The replication-defective rtM204V mutation was compensated by rtL180M mutation.

### 3.4. Susceptibility of Patient-Derived and BFV-Resistant Clones to Other Antiviral Agents

**Figure 4:**
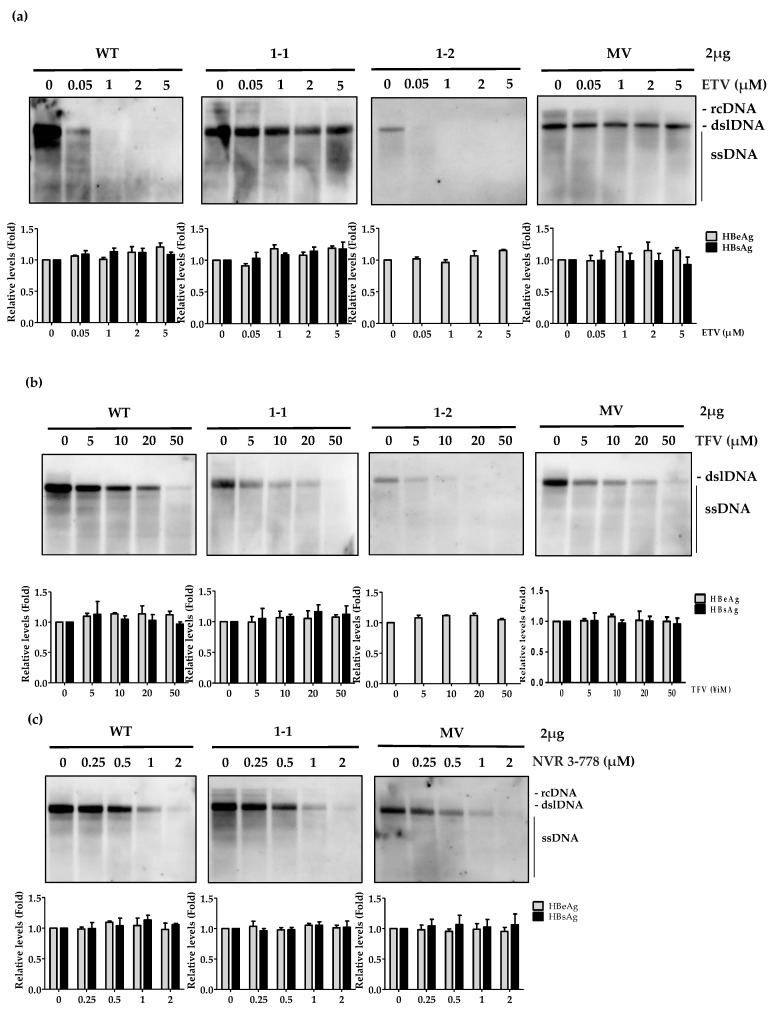
Susceptibility of BFV-resistant mutants to other antiviral drugs. (**a**,**b**) The in vitro drug susceptibility assay of patient-derived (1-1 and 1-2) and artificial MV clones was performed after treatment with ETV or TFV. After transfection with 2 µg of the indicated HBV 1.2mer mutants into Huh7 cells were treated with indicated antiviral agents (ETV or TFV) for 4 days. HBV replicative capacity was analyzed using Southern blot and detected with a DIG-labeled probe. The secreted HBV antigen levels (HBeAg, HBsAg) were analyzed by ELISA. (**c**) The drug susceptibility assay of patient-derived and artificial MV clones was performed after treatment with capsid assembly inhibitor, NVR 3-778. After transfection with indicated HBV 1.2 mer mutants into Huh7, cells were treated with NVR 3-778 for 4 days. HBV replicative capacity was analyzed using Southern blot.

Our results showed that the Clones 1-1 and MV were strongly resistant to BFV whereas Clone 1-2 was susceptible to BFV. Therefore, we tested whether these clones are susceptible to the most popular antiviral agents today, such as ETV and TFV. Clones 1-1 and MV exhibited resistance to ETV treatment (Figure 4a). This result is consistent to the previous reports that rtL180M and rtM204V mutations are associated with ETV resistance [28,29]. Clone 1-2 was susceptible to BFV as well as ETV.

However, these BFV-resistant clones were all susceptible to TFV treatment (Figure 4b). These results may explain the significant reduction of viremia in patient after changing antiviral treatment from BSV to TAF (Figure 1a). Similarly, we tested whether the BFV-resistant clones are susceptible to a capsid assembly modulator (NVR 3-778) [30,31]. Treatment with this non-polymerase targeting inhibitor strongly reduced the replication of BFV-resistant clones (Figure 4c).

Collectively, these findings suggest that TFV or capsid assembly modulators may be the options for treatment of CHB patients with BFV resistance.

## 4. Discussion

In this study, we identified a double BSV-resistant mutation in HBV isolated from one CHB patient with clinical resistance to BSV treatment and confirmed in vitro BSV resistance. The patient had a previous exposure to ETV but had a follow-up loss for more than 5 years, and viral breakthrough or HBV mutation were not previously demonstrated. To the best of our knowledge, this is the first report of HBV mutants with both clinical and in vitro resistance to BSV treatment.

The mechanism of BSV resistance in patient who was positive for rtL180M and rtM204V mutations is not clear. BSV is a nucleotide analogue, which might be theoretically effective against LAM-resistance HBV mutants [32], however in practice it could not inhibit the viral breakthrough. To date, there has been only one study in which BSV has been used in LAM-resistant CHB, thus information on BSV resistance and mutant virus fitness is relatively scarce [33]. In 2010, BSV was prescribed to 65 patients with LAM-resistant HBV, and it was effective at reducing viral load during 12 weeks of administration. In this study, the proportion of patients with L180M and M204V mutant HBV was 93.4% and 65.6% respectively [33]. The first difference between the previous report and our study is the duration of BSV treatment. In the previous study, after 12 weeks of BSV administration, drug regime was switched to ADV for up to 24 weeks. In our subject, resistance developed after 68 weeks of BSV treatment, which is much longer than the 12 weeks. Furthermore, in the previous study only the YMDD mutations were analyzed using the line probe assay (INNO-LiPA). This assay is relative not sensitive compared to our cloning and sequencing analysis, and cannot discriminate whether the two rtM204V and rtM204I mutations are separately or simultaneously exist in the same HBV genome. Also, there is a possibility that those patients harbor some mutations other than YMDD site. Those mutations in the non-YMDD site can affect the BSV resistance.

The results obtained from current study, suggest that long-term use of BSV in patients with LAM-resistance needs to be closely observed for the emerging resistant mutations.

In case of naïve CHB patients who administered BSV, drug resistance has not been found yet. In a phase 3 clinical trial compared to tenofovir, 11 patients experienced viral breakthrough in TDF-BSV switch group during the second year [18]. However, no drug resistant mutations were confirmed in these patients and HBV DNA went down to undetectable level at the follow-up visits in all patients.

NAs effectively inhibit HBV replication, attenuating liver damage and inflammation [8,9,34]. However, long-term treatment is inevitable as the inhibition of HBV replication disappears following drug withdrawal. Drug resistance and common side effects are major disadvantages of treatment with NAs in long-term. In addition, NAs target HBV RT rather than cccDNA which is the persistent form of viral DNA in the hepatocytes, therefore fundamental treatment is impossible. The BSV shows a strong HBV antiviral effect and is safer than TDF [13,21]. However, we found patients whom BSV intake failed to fully suppress HBV DNA level. The rtL180M and rtM204V mutations may have been occurred gradually while taking ETV as the first line anti-HBV agent. Even in drug naïve patient, the rtL180M and rtM204V might be selected as dominant mutant after long time treatment with BSV.

In this study HBV DNA was obtained from patient serum and sequenced after TA-cloning. Subsequently, the characteristic mutations (Clone 1-1 and 1-2) were identified by sequencing, and Clone 1-1 was resistant to BSV in vitro, whereas Clone 1-2 showed similar susceptibility to BFV as WT (Figure 1b). Additional experiments were conducted to find out which amino acid mutations in Clone 1-1 confers BSV resistance. As described in Materials and methods, six artificial replicons were generated from Clone 1-1 by infusion-cloning. After transfection of 1-1 mutation derived-replicons into human hepatocytes, BFV resistance and HBV replication ability were tested. As a result, artificial replicon with rtL180M and rtM204V mutations showed BFV resistance, proving that BFV resistance appears when rtL180M and/or rtM204V mutations are existed. On the other hand, rtL180M itself seems to be resistant to BFV as shown in Figure 3c, but there was stronger resistance to BFV in MV clone containing rtL180M and rtM204V simultaneously. Besides, as shown in Table 4, the IC_50_ value of the rtL180M mutant was more than 5 times than that of WT rendering partial-resistant to BFV. In addition, rtL180M is a representative mutation that shows resistance to various NAs. Based on RT-qPCR data, the rtM204V mutant IC_50_ value for BFV was more than 50 µM, meaning that it could induces resistance to BFV *per se*. However, combining PCR and Southern blot data, BFV resistance efficiently appeared only when rtL180M and rtM204V mutations exist together.

In in vitro experiments, rtM204V exhibited low replication capacity thus it is unlikely for rtL180M and rtM204V mutations to become a dominant variant itself. But it may become a dominant species when combined with rtL180M mutation in vivo because of enhanced replication capability. In line with our data (Figure 4a), the rtL180M and rtM204V substitutions have been reported in previous studies as ETV and LMV resistant mutations [24,27].

Due to the fact that the NAs, must be administered for a long time and have a high probability of developing resistance mutations, capsid assembly modulators (CpAMs) may be used as alternative method to overcome these issues. CpAMs inhibit the replication of HBV by suppressing the capsid formation in the HBV life-cycle. Among several types of CpAMs, NVR 3-778 belongs to class II CpAMs and prevents the virion production by promoting the formation of empty capsids [30,35]. Here, we examined the efficacy of NVR 3-778 on reducing the replication of drug resistant HBV mutants (Figure 4c) and provided evidence that capsid modulators could be accompanied by NAs to fully suppress HBV in CHB patients.

Despite the BSV resistance issue reported here and similar studies, for the treatment of naïve patients, BSV is still an effective high-barrier antiviral agent with low toxicity [36]. In phase II clinical trials, BSV showed same antiviral property compared with ETV over 96 weeks. In phase III clinical trial compared with TDF, BSV demonstrated durable, potent antiviral activity up to 192 weeks [18,37]. In this phase III clinical trial, BSV showed further improvement of bone mineral density and glomerular filtration rate, which were side effects of TDF. Furthermore, BSV therapy was reported to improve hepatic histology and decreased intrahepatic covalently closed circular DNA (cccDNA) in CHB patients [38]. In this study, at the time of the first BSV administration, ETV was discontinued for more than 5 years, and resistance had not been documented before, for this reason we selected BSV as the next antiviral regime for our subject. Of note, this patient had a history of exposure to antiviral drugs in the past, so even if there was no pre-existing resistance, it would have been better to choose an antiviral drug with more clinical evidence, such as TDF.

The biggest limitation of this study is that BSV resistance has been documented only in one patient. BSV has been on the market for less than 5 years, and is currently being sold officially only in Korea, thus the clinical experience is not yet sufficient compared to other drugs. Further in vitro and clinical experiments is required in order to understand the actual effect of BSV in other LAM-resistant patients.

Considering the fact that the clinical guidelines for multidrug-resistant (MDR) HBV strains and use of BSV in these patients are still limited, the value of current study is to raise awareness for physicians to suspect and identify the occurrence of BSV-resistant HBV mutants in CHB patients who reveal viral breakthrough despite good adherence to BSV-containing regimens.

In conclusion, although the use of BSV in our CHB subject was very successful for more than 60 weeks because of a high genetic barrier to resistance, we herein report that HBV isolated from a patient who developed phenotypic resistance during BSV therapy, showed in vitro genotypic resistance as well. Furthermore, to develop clinical resistance to BSV, the accumulation of at least two mutations was required. Accordingly, our findings suggest that caution should be exercised in using BSV as first-line therapy in patients with a history of LAM resistance.

## Figures and Tables

**Table 1:** Virological, serological, and biochemical characteristics of the study subject.

Variables	Values	Standard Levels
HBeAg	Positive	-
HBeAg antibody	Negative	-
HBV DNA (IU/mL)	6,954,754.3	-
bilirubin (mg/dL)	0.81	0.2~1.2
AST (IU/L)	66	5~40
ALT (IU/L)	101	0~40
Prothrombin time (s)	11.6	9.5~13.5
White blood cell (10^3^/μL)	5.46	4~10
Serum albumin (mg/dL)	4.5	3.3~5.2
Hemoglobin (g/dL)	13.6	12~16
Platelet (10^3^/μL)	157	150~450

**Table 2:** HBV RT mutations isolated from the BSV-treated patient.

HBV Clone Wild-Type (GQ872210)	Amino Acid Position																								
	23	38	54	55	68	110	123	124	134	139	180	191	204	207	226	244	266	267	269	285	303	317	329	333	336
	V	T	T	H	S	R	N	Y	D	N	L	V	M	V	N	G	V	Q	L	K	C	S	A	K	L
1-1,14,19,21,22	I			R				H	E	K	M		V					L	I						M
1-2,16		A										I			H		I	L				A	T		
1-15,24						G	D	H										L	I					Q	
1-23,30																									
1-3	I		S	R				H	E	K	M		V					L	I						M
1-4	I			R				H	E	K	M		V	L				L	I						M
1-17					P																				
1-25																S									
1-31						G	D	H										L	I	R	R			Q	

Columns with gray background highlight representative besifovir-resistant mutation (IRHEKMVLIM).

**Table 3:** Patient-derived common mutations in HBV RT and overlapping surface antigen.

Clone	Region	Mutation in the Corresponding Gene																			
**WT**	**RT**	V 23	T 38	T 42	H 55	L 102	Y 124	D 134	N 139	Y 141	G 165	L 180	V 191	M 204	N 226	V 266	Q 267	L 269	S 317	A 329	L 336
	**S**	L 15	Q 30	S 34	T 47	L 94	T 116	I 126	T 131	M 133	A 157	W 172	W 182	I 195	I 218	-	-	-	-	-	-
**1-1**	**RT**	I			R		H	E	K			M		V			L	I			M
	**S**			L	A			S	N	T				M							
**1-2**	**RT**		A										I		H	I	L		A	T	
	**S**					S		T	T		D	*	*								

* Stop codon.

**Table 4:** Replication ability and IC_50_ values of mutant clones against BFV in Huh7 cells.

Clone	Replication Ability (%)	IC_50_ (μM)	Fold Resistance (/WT IC_50_)
WT	100	4.13 ± 0.52	1.00
1-1	22.36 ± 0.08	>50	>12.1
1-2	3.75 ± 1.59	3.83 ± 0.6	0.92
M	58.84 ± 0.82	23.87 ± 4.07	5.8
V	4.6 ± 0.39	>50	>12.1
MV	12.21 ± 1.02	>50	>12.1

The quantitative real-time PCR experiment was performed in three independent biological replicates.

## Data Availability

Not applicable.

## Supplementary Materials

The following supporting information can be downloaded at: https://www.mdpi.com/article/10.3390/biomedicines10020282/s1, Figure S1: The MV mutation causing BFV resistance in 1-1 clones was shown by SANGER sequencing; Table S1: The list of primer sequences used in this study.

## References

[1] Sun Y., Wang S., Yi Y., Zhang J., Duan Z., Yuan K., Liu W., Li J., Zhu Y. (2018). The Hepatitis B Surface Antigen Binding Protein: An Immunoglobulin G Constant Region-Like Protein That Interacts With HBV Envelop Proteins and Mediates HBV Entry. Front. Cell. Infect. Microbiol..

[2] Shaw T., Bartholomeusz A., Locarnini S. (2006). HBV drug resistance: Mechanisms, detection and interpretation. J. Hepatol..

[3] Clark D.N., Hu J. (2015). Unveiling the roles of HBV polymerase for new antiviral strategies. Future Virol..

[4] Tu T., Budzinska M.A., Vondran F.W.R., Shackel N.A., Urban S. (2018). Hepatitis B Virus DNA Integration Occurs Early in the Viral Life Cycle in an In Vitro Infection Model via Sodium Taurocholate Cotransporting Polypeptide-Dependent Uptake of Enveloped Virus Particles. J. Virol..

[5] Ko C., Chakraborty A., Chou W.M., Hasreiter J., Wettengel J.M., Stadler D., Bester R., Asen T., Zhang K., Wisskirchen K. (2018). Hepatitis B virus genome recycling and de novo secondary infection events maintain stable cccDNA levels. J. Hepatol..

[6] Dusseaux M., Masse-Ranson G., Darche S., Ahodantin J., Li Y., Fiquet O., Beaumont E., Moreau P., Riviere L., Neuveut C. (2017). Viral Load Affects the Immune Response to HBV in Mice With Humanized Immune System and Liver. Gastroenterology.

[7] Konig A., Yang J., Jo E., Park K.H.P., Kim H., Than T.T., Song X., Qi X., Dai X., Park S. (2019). Efficient long-term amplification of hepatitis B virus isolates after infection of slow proliferating HepG2-NTCP cells. J. Hepatol..

[8] Yasutake Y., Hattori S.I., Tamura N., Matsuda K., Kohgo S., Maeda K., Mitsuya H. (2020). Structural features in common of HBV and HIV-1 resistance against chirally-distinct nucleoside analogues entecavir and lamivudine. Sci. Rep..

[9] Seifer M., Patty A., Serra I., Li B., Standring D.N. (2009). Telbivudine, a nucleoside analog inhibitor of HBV polymerase, has a different in vitro cross-resistance profile than the nucleotide analog inhibitors adefovir and tenofovir. Antivir. Res..

[10] Kim K.H., Kim N.D., Seong B.L. (2010). Discovery and development of anti-HBV agents and their resistance. Molecules.

[11] Kwon S.Y., Park Y.K., Ahn S.H., Cho E.S., Choe W.H., Lee C.H., Kim B.K., Ko S.Y., Choi H.S., Park E.S. (2010). Identification and characterization of clevudine-resistant mutants of hepatitis B virus isolated from chronic hepatitis B patients. J. Virol..

[12] Korean Association for the Study of the Liver (2019). KASL clinical practice guidelines for management of chronic hepatitis B. Clin. Mol. Hepatol..

[13] Ahn S.H., Kim W., Jung Y.K., Yang J.M., Jang J.Y., Kweon Y.O., Cho Y.K., Kim Y.J., Hong G.Y., Kim D.J. (2019). Efficacy and Safety of Besifovir Dipivoxil Maleate Compared With Tenofovir Disoproxil Fumarate in Treatment of Chronic Hepatitis B Virus Infection. Clin. Gastroenterol. Hepatol. Off. Clin. Pract. J. Am. Gastroenterol. Assoc..

[14] Lai C.L., Ahn S.H., Lee K.S., Um S.H., Cho M., Yoon S.K., Lee J.W., Park N.H., Kweon Y.O., Sohn J.H. (2014). Phase IIb multicentred randomised trial of besifovir (LB80380) versus entecavir in Asian patients with chronic hepatitis B. Gut.

[15] Jung J.A., Kim S.R., Kim T.E., Kim J.R., Lee S.Y., Huh W., Ko J.W. (2012). Pharmacokinetic comparison of the maleate and free base formulations of LB80380, a novel nucleotide analog, in healthy male volunteers. Int. J. Clin. Pharmacol. Ther..

[16] Fung J., Lai C.L., Yuen M.F. (2008). LB80380: A promising new drug for the treatment of chronic hepatitis B. Expert Opin. Investig. Drugs.

[17] Yuen M.F., Ahn S.H., Lee K.S., Um S.H., Cho M., Yoon S.K., Lee J.W., Park N.H., Kweon Y.O., Sohn J.H. (2015). Two-year treatment outcome of chronic hepatitis B infection treated with besifovir vs. entecavir: Results from a multicentre study. J. Hepatol..

[18] Song D.S., Kim W., Ahn S.H., Yim H.J., Jang J.Y., Kweon Y.O., Cho Y.K., Kim Y.J., Hong G.Y., Kim D.J. (2021). Continuing besifovir dipivoxil maleate versus switching from tenofovir disoproxil fumarate for treatment of chronic hepatitis B: Results of 192-week phase 3 trial. Clin. Mol. Hepatol..

[19] Lee A.R., Cho J.Y., Kim J.C., Dezhbord M., Choo S.Y., Ahn C.H., Kim N.Y., Shin J.J., Park S., Park E.S. (2021). Distinctive HBV Replication Capacity and Susceptibility to Tenofovir Induced by a Polymerase Point Mutation in Hepatoma Cell Lines and Primary Human Hepatocytes. Int. J. Mol. Sci..

[20] Lucifora J., Salvetti A., Marniquet X., Mailly L., Testoni B., Fusil F., Inchauspe A., Michelet M., Michel M.L., Levrero M. (2017). Detection of the hepatitis B virus (HBV) covalently-closed-circular DNA (cccDNA) in mice transduced with a recombinant AAV-HBV vector. Antivir. Res..

[21] Park E.S., Lee A.R., Kim D.H., Lee J.H., Yoo J.J., Ahn S.H., Sim H., Park S., Kang H.S., Won J. (2019). Identification of a quadruple mutation that confers tenofovir resistance in chronic hepatitis B patients. J. Hepatol..

[22] Park S., Park E.S., Koo J.E., Park Y.K., Lee A.R., Dezhbord M., Cho E.S., Ahn S.H., Kim D.H., Lee J.H. (2020). Entecavir-resistant hepatitis B virus decreases surface antigenicity: A full genome and functional characterization. Liver Int..

[23] Li M.W., Hou W., Wo J.E., Liu K.Z. (2005). Character of HBV (hepatitis B virus) polymerase gene rtM204V/I and rtL180M mutation in patients with lamivudine resistance. J. Zhejiang Univ. Sci. B.

[24] Nakajima S., Watashi K., Kato T., Muramatsu M., Wakita T., Tamura N., Hattori S.I., Maeda K., Mitsuya H., Yasutake Y. (2021). Biochemical and Structural Properties of Entecavir-Resistant Hepatitis B Virus Polymerase with L180M/M204V Mutations. J. Virol..

[25] Pal A., Sarkar N., Saha D., Guha S.K., Saha B., Chakrabarti S., Chakravarty R. (2015). High incidence of lamivudine-resistance-associated vaccine-escape HBV mutants among HIV-coinfected patients on prolonged antiretroviral therapy. Antivir. Ther..

[26] Liu Y., Zhou Y., Li X., Niu M., Chen R., Shao J., Si L., Luo D., Lin Y., Li L. (2019). Hepatitis B virus mutation pattern rtL180M+A181C+M204V may contribute to entecavir resistance in clinical practice. Emerg. Microbes Infect..

[27] Wang Y., Liu S., Chen Y.U., Zheng S., Zhou L.I., Lu F., Duan Z. (2016). Lamivudine-resistant rtL180M and rtM204I/V are persistently dominant during combination rescue therapy with entecavir and adefovir for hepatitis B. Exp. Ther. Med..

[28] Suzuki F., Sezaki H., Hosaka T., Suzuki Y., Fujiyama S., Kawamura Y., Akuta N., Kobayashi M., Saitoh S., Arase Y. (2021). Virologic analysis of tenofovir resistance in a patient with chronic hepatitis B experiencing viral breakthrough during combination treatment with tenofovir disoproxil fumarate and entecavir. Hepatol. Res..

[29] Tenney D.J., Rose R.E., Baldick C.J., Pokornowski K.A., Eggers B.J., Fang J., Wichroski M.J., Xu D., Yang J., Wilber R.B. (2009). Long-term monitoring shows hepatitis B virus resistance to entecavir in nucleoside-naive patients is rare through 5 years of therapy. Hepatology.

[30] Yuen M.F., Gane E.J., Kim D.J., Weilert F., Yuen Chan H.L., Lalezari J., Hwang S.G., Nguyen T., Flores O., Hartman G. (2019). Antiviral Activity, Safety, and Pharmacokinetics of Capsid Assembly Modulator NVR 3-778 in Patients with Chronic HBV Infection. Gastroenterology.

[31] Zhao Q., Hu Z., Cheng J., Wu S., Luo Y., Chang J., Hu J., Guo J.T. (2018). Hepatitis B Virus Core Protein Dephosphorylation Occurs during Pregenomic RNA Encapsidation. J. Virol..

[32] Tseng T.C. (2021). Another oral antiviral treatment, but still far away from hepatitis B virus cure. Clin. Mol. Hepatol..

[33] Yuen M.F., Han K.H., Um S.H., Yoon S.K., Kim H.R., Kim J., Kim C.R., Lai C.L. (2010). Antiviral activity and safety of LB80380 in hepatitis B e antigen-positive chronic hepatitis B patients with lamivudine-resistant disease. Hepatology.

[34] You C.R., Lee S.W., Jang J.W., Yoon S.K. (2014). Update on hepatitis B virus infection. World J. Gastroenterol..

[35] Lam A.M., Espiritu C., Vogel R., Ren S., Lau V., Kelly M., Kuduk S.D., Hartman G.D., Flores O.A., Klumpp K. (2019). Preclinical Characterization of NVR 3-778, a First-in-Class Capsid Assembly Modulator against Hepatitis B Virus. Antimicrob. Agents Chemother..

[36] Song J.E., Park J.Y. (2021). Besifovir dipivoxil maleate: A novel antiviral agent with low toxicity and high genetic barriers for chronic hepatitis B. Expert Opin. Pharm..

[37] Yim H.J., Kim W., Ahn S.H., Yang J.M., Jang J.Y., Kweon Y.O., Cho Y.K., Kim Y.J., Hong G.Y., Kim D.J. (2020). Besifovir Dipivoxil Maleate 144-Week Treatment of Chronic Hepatitis B: An Open-Label Extensional Study of a Phase 3 Trial. Am. J. Gastroenterol.

[38] Yim H.J., Kim W., Ahn S.H., Jung Y.K., Um S.H., Sohn J.H., Jang J.Y., Kim D.J., Park E.S., Jin S.Y. (2021). Besifovir Therapy Improves Hepatic Histology and Reduces Covalently Closed Circular DNA in Chronic Hepatitis B Patients. J. Gastroenterol. Hepatol..

