# Supplementary material for: Identification and Characterization of Besifovir-Resistant Hepatitis B Virus Isolated from a Chronic Hepatitis B Patient
Source: Biomedicines. 2022 Jan 26;10(2):282. doi: 10.3390/biomedicines10020282 (PMC8868672; doi:10.3390/biomedicines10020282)
Supplement: Supplementary file 1 [file biomedicines-10-00282-s001.zip › biomedicines-1509954-Supplementary.pdf]

Supplementary files

Supplementary Table S1. The list of primer sequences used in this study.

Clone	Fragment	Primer	Sequence (5'-3')
IRHEK	Fragment 1	Forward	CTC CCT TAT CGT CAA TCT TCT CGA GAC TGG GGA CCC TG
		Reverse	AAC AAG AGG GAA ACA TAG AGT TTC CTT GAG CAG GAC TCG
	Fragment 2	Forward	CTC TAT GTT TCC CTC TTG TTG CTG TAC AAA ACC TTC GGA
		Reverse	TGG CAG CAC AGC CTA GCA GCC ATG GGA AGG AGG TGT ATT TCC G
MVLIM	Fragment 1	Forward	GAG TGG GCC TCA GTC CGT TTC TCA TGG CTC AGT TTA CTA
		Reverse	TGG CAG CAC AGC CTA GCA GCC ATG GGA AGG AGG TGT ATT TCC G
	Fragment 2	Forward	CTC CCT TAT CGT CAA TCT TCT CGA GAC TGG GGA CCC TG
		Reverse	AAA CGG ACT GAG GCC CAC TCC CAT AGG AAT CTT GCG AAA G
LIM	Fragment 1	Forward	GAT ATG TAA TTG GAA GTT GGG GTA CTT TAC CAC AGG AAC A
		Reverse	TGG CAG CAC AGC CTA GCA GCC ATG GGA AGG AGG TGT ATT TCC G
	Fragment 2	Forward	CTC CCT TAT CGT CAA TCT TCT CGA GAC TGG GGA CCC TG
		Reverse	CCA ACT TCC AAT TAC ATA TCC CAT GAA GTT AAG GGA GTA G
M	Fragment 1	Forward	CTC CCT TAT CGT CAA TCT TCT CGA GAC TGG GGA CCC TG
		Reverse	GAA CAA ATG GCA CTA GTA AAC TGA GCC ATG AGA AAC GGA CT
	Fragment 2	Forward	GTT TAC TAG TGC CAT TTG TTC AGT GGT TCG CAG GGC TTT
		Reverse	TGG CAG CAC AGC CTA GCA GCC ATG GGA AGG AGG TGT ATT TCC G
V	Fragment 1	Forward	CTC CCT TAT CGT CAA TCT TCT CGA GAC TGG GGA CCC TG
		Reverse	ACT TGG CCC CCA ATA CCA CAT CAT CCA CAT AAC TGA AAG CC
	Fragment 2	Forward	TGT GGT ATT GGG GGC CAA GTC TGT ACA ACA TCT TGA GTC CC
		Reverse	TGG CAG CAC AGC CTA GCA GCC ATG GGA AGG AGG TGT ATT TCC G
HBV RT		Forward	AAT CTT CTC GAG GAC TGG GGA CCC TGC ACC
		Reverse	GAG CAG CCA TGG GAA GGA GGT GTA TTT CCG
Vector (HBV 1.2mer)		Forward	GCT GCT AGG CTG TGC TGC CAA C
		Reverse	GAA GAT TGA CGA TAA GGG AGA GGC AGT AG
RT-qPCR		Forward	CTC GTG GTG GAC TTC TCT C
		Reverse	CTG CAG GAT GAA GAG GAA

Supplementary Figure S1. The MV mutation causing BFV resistance in 1-1 clones was shown by SANGER sequencing.

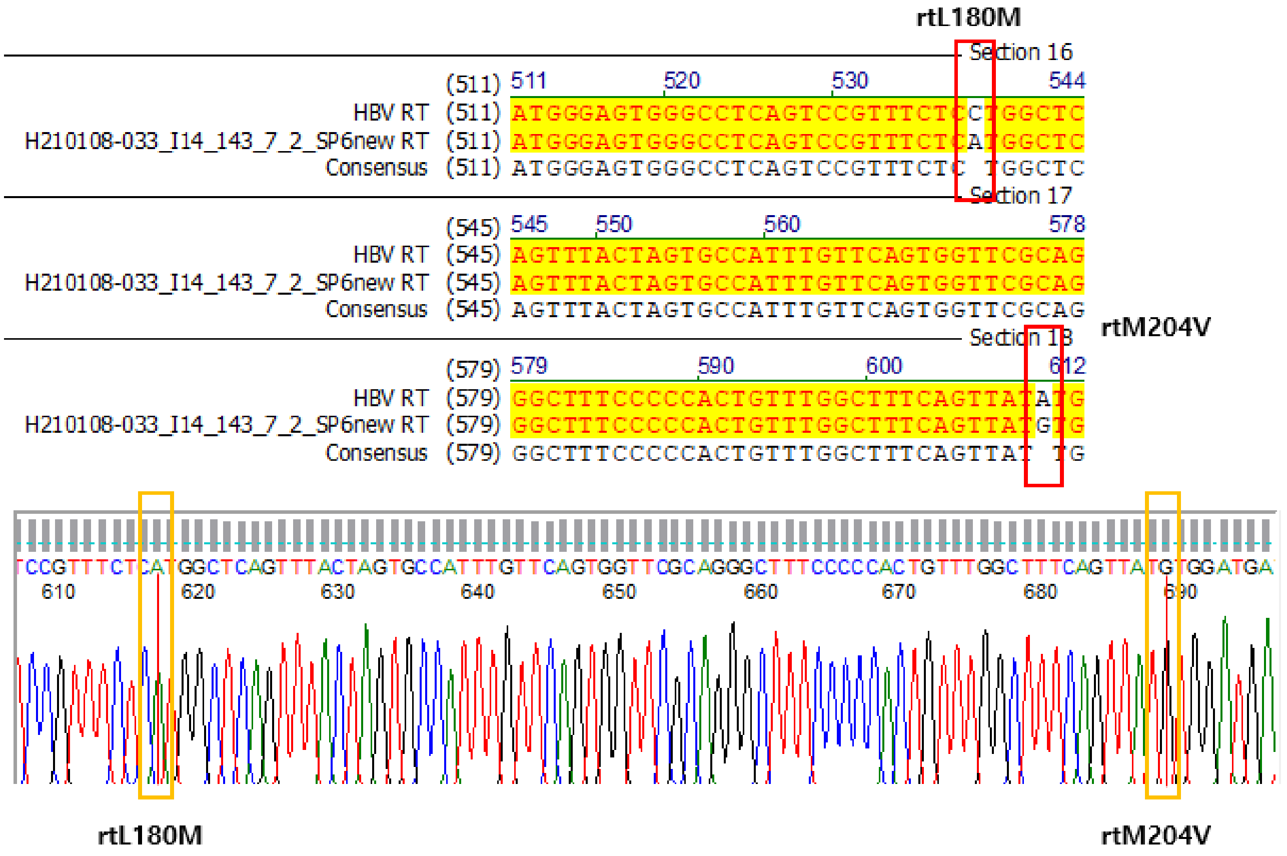

Upper electropherogram; the sequencing data, WT clone is shown as HBV RT and the 1-1 clone is shown as 143-7-2. At the positions 538 and 610, C and A in WT has been changed to A and G in clone 1-1 respectively. As a result of nucleotide substitution at 538 bp, the amino acid sequence has been altered from Leu in WT to Met in clone 1-1. The bp substitution at position 610 led to the amino acid alteration from Met in WT to Val in clone 1-1.

Bottom; BFV associated mutations has been highlighted.
